# Proteomics of *Bordetella pertussis* whole-cell and acellular vaccines

**DOI:** 10.1186/s13104-019-4373-2

**Published:** 2019-06-10

**Authors:** Jens Möller, Max Edmund Kraner, Andreas Burkovski

**Affiliations:** 1 Friedrich-Alexander-Universität Erlangen-Nürnberg, Staudtstr. 5, 91058 Erlangen, Germany; 2 Lehrstuhl für Biochemie, Friedrich-Alexander-Universität Erlangen-Nürnberg, Erlangen, Germany

**Keywords:** *Bordetella*, DTP vaccine, LC–MS/MS, Mass spectrometry, Pertussis, Vaccination, Whooping cough

## Abstract

**Objectives:**

*Bordetella pertussis* is the etiological agent of whooping cough, a bacterial infection of especially children, which may be fatal without treatment. In frame of studies to investigate putative effects of vaccination on host–pathogen interaction and clonal distribution of strains, in addition to *Corynebacterium diphtheriae* and *Clostridium tetani* toxoid vaccines, also whole-cell and acellular pertussis vaccines were analyzed by mass spectrometry.

**Data description:**

LC–MS/MS spectra were generated and analyzed using *B. pertussis* genome data and proteins present in whole-cell and acellular pertussis vaccines were identified. Subcellular localization of proteins and presence of signal peptides was determined bioinformatically.

## Objective

*Bordetella pertussis* is the etiological agent of whooping cough, a bacterial infection of especially children, which may be fatal without treatment. In 2008 about 16 million cases of whooping cough—also designated as pertussis—resulting in almost 200,000 pediatric deaths were estimated by the World Health Organization, while for 2015 more than 60,000 deaths of infants were assumed [1–3]. These numbers of worldwide cases illustrate that pertussis is a continuing threat to children’s health and vaccination is important to prevent this infection. In frame of studies to investigate putative effects of vaccination on host–pathogen interaction and clonal distribution of strains, in addition to *Corynebacterium diphtheriae* [4] and *Clostridium tetani* [5] toxoid vaccines, also pertussis vaccines were analyzed by mass spectrometry (MS). In order to identify putative immunogenic proteins and to provide data to understand the influence of vaccination on the distribution of *B. pertussis* strains, two different types of vaccines were analyzed in respect to their protein content: a whole-cellular and an acellular pertussis vaccine. Whole-cell vaccines are suspensions of killed *B. pertussis* cells, while the acellular pertussis vaccines, which were developed to prevent unwanted local reactions of the whole-cell vaccine, contain only a limited number of purified components (for overview of different vaccine formulations see [3]. An influence of vaccination strategy used and components included in the vaccine on evolution of *B. pertussis* strains in human populations was already discussed [6, 7], based on an observed effect of vaccination on the distribution of different alleles in *B. pertussis*. For example, the pertactin-encoding *prn1* allele was dominating in the pre-vaccine era, while after introduction of the whole-cellular vaccine produced with a *prn1*-carrying strain, *B. pertussis* isolates harboring the *prn2* allele increased [8]. Also a change in the fimbrial serotype of *B. pertussis* strains was observed from serotype Fim2 predominantly found in unvaccinated populations to serotype Fim3 and serotype Fim2,3 in vaccinated populations [9]. Since proteome analyses of *B. pertussis* and corresponding vaccines are scarce [10], the proteomics data presented in this data note may be helpful for further evolutionary studies.

## Data description

The data represent proteomic analyses of commercially vaccines, an acellular and a whole cellular pertussis vaccine. Raw data files (.raw), MS/MS spectra files (.msf), peptide sequence assignment files of MS/MS scans (pep.xml) as well as a list of identified proteins (.xlsx) were deposited to the ProteomeXchange Consortium (http://proteomecentral.proteomexchange.org) via the PRIDE partner repository [11] and are available with identifier PXD013804 (Table 1). The data set provided includes a table with identified proteins in the analyzed vaccines, their molecular function and cellular localization as well as a PDF file containing three images depicting these results, a list with vaccines used in this study and a table with identified proteins related to pathogenicity (Table 1).

**Table 1:** Overview of data files/data sets

Label	Name of data file/data set	File types (file extension)	Data repository and identifier (DOI or accession number)
Data set 1 [15]	*Bordetella pertussis* vaccine project	RAW data file (.raw), MS/MS spectra files (.msf), MS Excel file (.xlsx), peptide sequence assignment files (pep.xml)	http://identifiers.org/pride.project:PXD013804
Data set 2 [16]	Results table	MS Excel file (.xlsx)	10.6084/m9.figshare.8108165
Data set 3 [17]	Images of *Bordetella pertussis* vaccine analysis	PDF (.pdf)	10.6084/m9.figshare.8108210

### Methodology

#### Protein sample preparation

*Bordetella pertussis* vaccines are typically administered in combination with diphtheria and tetanus toxoid vaccines as DTP3 vaccines. In this study, we analyzed one acellular and one whole cellular commercially available pertussis vaccine (for details, see Data set 1). The sample preparation of the vaccines for mass spectrometry analysis was carried out as described recently [4, 5]. In short, the proteins were transferred to 10 kDa membrane filters and processed using a modified protocol for Filter Aided Sample Preparation (FASP) [12, 13]. After filter-based tryptic digest and elution, the peptides were desalted and concentrated using C18 stage tips. Prior to LC–MS/MS (liquid chromatography tandem–mass spectrometry), peptides were vacuum dried and solved in 0.1% trifluoroacetic acid (TFA) [14].

#### Mass spectrometry and data analysis

Mass spectrometric analyses were carried out as described before [4, 5, 13, 14] using a combination of a nanoflow Ultimate 3000 HPLC (Dionex, Sunnyvale, CA, USA) and an Orbitrap Fusion mass spectrometer (Thermo Fisher Scientific, Waltham, MA, USA). Resulting raw data files were analyzed using the *C. diphtheriae* ATCC 700971/NCTC 13129/Biotype gravis database (Proteome Id: UP000002198), the *Clostridium tetani* E88 database, (Proteome Id: UP000001412) and the *Bordetella pertussis* (strain Tohama I/ATCC BAA-589/NCTC 13251) database (Proteome Id.: UP000002676) using the Proteome Discoverer 1.4 program package (Thermo Fisher Scientific, Bremen, Germany). Identification of product ions was carried out as described recently [4, 5]: theoretical peptide masses for trypsin digestion were generated allowing two missed cleavages and the following settings were used for analysis: (i) carbamidomethyl modification of cysteine residues fixed, (ii) oxidation of methionine dynamic, (iii) mass tolerance 10 ppm for survey scans, (iv) 0.6 Da for fragment mass measurements, (v) protein identification threshold 1% false discovery rate (FDR).

In total 1855 unique proteins were identified by mass spectrometry analyses with 1850 distinct proteins identified in the whole cellular vaccine and 25 unique identified in the acellular vaccine.

## Limitations

The data presented here were obtained in frame of a project focusing on diphtheria and tetanus toxoid vaccines. Since these vaccines are administered in different combinations, only part of the samples contained *B. pertussis* proteins and the number of pertussis vaccines was limited to three.

## Data Availability

The data described in this Data note can be freely and openly accessed on (http://proteomecentral.proteomexchange.org) via the PRIDE partner repository and (https://figshare.com/). Please see Table 1 and reference list [15–17] for details and links to the data.

## Abbreviations

FASP: Filter Aided Sample Preparation
FDR: false discovery rate
LC–MS/MS: liquid chromatography tandem–mass spectrometry
MS: mass spectrometry
TFA: trifluoroacetic acid
WHO: World Health Organization
